# Chinese Herbal Formula Huayu-Qiangshen-Tongbi Decoction Compared With Leflunomide in Combination With Methotrexate in Patients With Active Rheumatoid Arthritis: An Open-Label, Randomized, Controlled, Pilot Study

**DOI:** 10.3389/fmed.2020.00484

**Published:** 2020-09-04

**Authors:** Jiaqi Wu, Xianghong Chen, Yuan Lv, Kaixin Gao, Zehao Liu, Yue Zhao, Xiumin Chen, Xiaohong He, Yongliang Chu, Xiaodong Wu, Aihua Ou, Zehuai Wen, Jianyong Zhang, Jianhong Peng, Zhisheng Huang, Per-Johan Jakobsson, Qingchun Huang, Runyue Huang

**Affiliations:** ^1^The Second Affiliated Hospital of Guangzhou University of Chinese Medicine (Guangdong Provincial Hospital of Chinese Medicine), Guangzhou, China; ^2^Second Clinical Medical College, Guangzhou University of Chinese Medicine, Guangzhou, China; ^3^Ruikang Hospital Affiliated to Guangxi University of Chinese Medicine, Guangxi, China; ^4^Guangdong Provincial Key Laboratory of Clinical Research on Traditional Chinese Medicine Syndrome, and State Key Laboratory of Dampness Syndrome of Chinese Medicine, The Second Affiliated Hospital of Guangzhou University of Chinese Medicine, Guangzhou, China; ^5^Shenzhen Hospital of Traditional Chinese Medicine, Shenzhen, China; ^6^Dongguan Hospital of Traditional Chinese Medicine, Dongguan, China; ^7^Guangzhou Hospital of Integrated Traditional Chinese and Western Medicine, Huadu, China; ^8^Rheumatology Unit, Department of Medicine Solna, Karolinska Institutet, Karolinska University Hospital, Stockholm, Sweden

**Keywords:** combination therapy, Chinese medical formula, Huayu-Qiangshen-Tongbi decoction, randomized controlled clinical trial, pilot study

## Abstract

**Background:** Traditional Chinese Medicine is complementary and an alternative to modern medicine. The combination therapies of herbal products with disease-modifying anti-rheumatic drugs are gradually and widely adopted in the management of rheumatoid arthritis (RA) in China.

**Purpose:** To evaluate the efficacy and safety of Huayu-Qiangshen-Tongbi (HQT) decoction, a Chinese medicine formula, combined with methotrexate (MTX) in the treatment of patients with active RA, in comparison with the combination therapy of MTX with leflunomide (LEF).

**Methods:** This pilot study was a monocenter, open-label, randomized controlled trial with two parallel arms. Ninety patients with active RA were randomly allocated to receive either HQT at a dose of 250 ml twice daily or LEF at a dose of 20 mg once daily, and all participants received MTX at a dose of 10–15 mg once weekly. The primary efficacy endpoint was the proportion of patients who achieved a 20% improvement in the American College of Rheumatology criteria (ACR20) after a 24-week treatment.

**Results:** 84.4% (76/90) patients completed the 24-week observation. In the intention-to-treat analysis, the percentage values of patients achieving the ACR20 response criteria were 72.1% (31/43) in MTX + HQT group and 74.4% (32/43) in MTX + LEF group (*p* = 0.808). No significant difference was observed in other parameters, including ACR50, ACR70, clinical disease activity index good responses, European League Against Rheumatism good response, remission rate, and low disease activity rate. The results of the per-protocol analysis showed consistency with those of the intention-to-treat analysis. The mean change from baseline at week 24 for the van der Heijde modified total sharp score had no significant difference between two groups (3.59 ± 4.75 and 1.34 ± 8.67 in the MTX + HQT group and MTX + LEF group, respectively, *p* = 0.613). The frequency of adverse events was similar in both groups (11 cases in the MTX + HQT and 17 cases in the MTX + LEF, *p* > 0.05).

**Conclusions:** In patients with active RA, treatment with the combination of HQT and MTX was associated with improvement in signs, symptoms, and physical function. With a beneficial clinical response and acceptable tolerability, HQT or other Chinese medicine formula may be a good therapeutic option in combination with MTX for RA treatment.

**Trial registration:** Chinese Clinical Trails Registry, ChiCTR-INR-16009031, Registered on 15th August 2016, http://www.chictr.org.cn/enindex.aspx.

## Introduction

Rheumatoid arthritis (RA) is a common autoimmune musculoskeletal disease affecting the joints primarily, leads to structural damage including cartilage destruction and bone erosion, and brings about extra-articular harm such as cardiovascular, pulmonary, and psychological disorders (1). Disease-modifying anti-rheumatic drugs (DMARDs) are the principal choice of first-line treatments for patients with RA, among which methotrexate (MTX) is well-established as an anchor drug for both treatment and research (2). However, not all patients receiving MTX monotherapy achieved low disease activity (LDA) or clinical remission (3). Over the last two decades, the treatment of RA has been transformed, and today, in patients with insufficient response to MTX monotherapy, combination with biological DMARDs or other conventional synthetic DMARDs (csDMARDs) is an international consensus of RA therapeutic strategy (2). Although new effective treatment regimens increased the clinical response rate of achieving full or long-lasting remission, a substantial number of RA patients did not respond to the current therapeutic strategies and even suffered from adverse effects (AEs) caused by long-term treatments, such as gastrointestinal toxicity, hepatotoxicity, bone marrow suppression, tuberculosis, and infection (2, 4). Previous researches reported that the use of prednisone and certain biological DMARDs increased the risk of tuberculosis and other opportunistic infections occurring in RA patients (4–6). Due to the development of advance effects, a portion of RA patients did not benefit from these combination therapeutic regimens and discontinued the treatment (7, 8). Therefore, there is still a considerable unmet need in RA treatment, and an application of new effective and safe treatment strategies should now be the priority of research efforts.

Traditional Chinese medicine (TCM), such as herbal products and acupuncture, has been widely practiced in clinics for over thousands of years in China and has found to be effective in treating many types of diseases, such as RA. Several Chinese medical herbs and their formulas, extracts, active ingredients, and even single compounds have been used for the RA treatment. Their clinical efficacy against RA and the safety have been evidenced by clinical practices and clinical trials in RA patients (9–12). Huayu-Qiangshen-Tongbi decoction (HQT) is a Chinese medical formula used in RA treatment in Guangdong Provincial Hospital of Chinese Medicine, which is composed of the following natural materials: the root and rhizoma of *Salvia miltiorrhiza* Bunge (Danshen), the rhizoma of *Dioscorea nipponica* Makino (Chuanshanlong), the root of *Astragalus membranaceus* (Huangqi), the root of *Paeonia tacti lora Pall* (Baishao), the root, stem, and leaf of *Saussurea involucrata* (Kar. et Kir.) Sch.-Bip (Tianshanxuelian), the bark of *Eucommia ulmoides* Oliver (Duzhong), the root and rhizoma of *Davallia mariesii* Moore ex Bak (Gusuibu), the root of *Dipsacus asperoides* C. Y. Cheng et T. M. Ai (Chuanxuduan), the earthnut of Chinese Foxglove (Shudi), and the root and rhizoma of *Glycyrrhiza uralensis* (Gancao). In our hospital, HQT has been used for RA management with the combination of csDMARDs, especially the MTX. Notably, we had undertaken a retrospective record review to evaluate the clinical response and AEs of the combination therapy of HQT and MTX in 2019. The result of the retrospective study showed that HQT combined with MTX had favorable therapeutic effects in improving the overall symptoms of RA patients with good tolerance (13). HQT may function as a kind of DMARDs, which can be used as an alternative or add-on treatment against RA. The purpose of this study is to determine the efficacy and safety of HQT in combination with MTX by performing an investigator-initiated, 24-week prospective, randomized clinical study, which might provide basic data and evidence for a further undergoing multicenter, double-blinded, randomized, placebo-controlled trial.

## Methods

### Study Design

This pilot study was a 24-week, monocenter, open-label, randomized controlled trial, which was conducted in the Second Affiliated Hospital of Guangzhou University of Chinese Medicine (Guangdong Provincial Hospital of Chinese Medicine) between August 2016 and September 2018. All the participants were provided written informed consent, and the protocol was first approved by the Medical Ethics Committee of the Second Affiliated Hospital of Guangzhou University of Chinese Medicine (B2016-076-01) and subsequently registered with the World Health Organization clinical trial registry (no. ChiCTR-INR-16009031).

### Patients

All participants were recruited from an outpatient rheumatology clinic at the Second Affiliated Hospital of Guangzhou University of Chinese Medicine (Guangdong Provincial Hospital of Chinese Medicine) in Guangzhou, China. Individuals with RA were all screened in clinics based on the inclusion and exclusion criteria, which are described later. If the eligibility criteria were met, the patients would be asked if they were interested in participating in the trial. The trial coordinator contacted participants to explain the requirements and purpose of the study, and the informed consent was completed as well.

### Inclusion/Exclusion Criteria

Participants should meet the following criteria in this study: (1) aged between 18 and 65 years; (2) diagnosed with RA based on the diagnostic criteria of 1987 American College of Rheumatology (ACR) (14) or the 2009 ACR criteria (15); (3) were in functional class I, II, or III (according to the 1987 American Rheumatism Association classification standard) (14); (5) Chinese medicine inclusion criteria: with a syndrome pattern including wind and damp stagnation, cold and damp stagnation, hot and damp stagnation, phlegm and stasis stagnation, and deficiency of kidney and liver (criteria of Chinese medicine symptoms assessment is shown in Appendix 1); (5) the 28-joint disease activity score (DAS) based on C-reactive protein (DAS28-CRP) score > 3.2 (16); (6) received a stable dose of non-steroidal anti-inflammatory drug (NSAID) during the 4 weeks before screening, or did not take NSAIDs before screening for at least 1 week; (7) did not take DMARDs (including biological DMARDs and csDMARDs) before screening during the 4 weeks; the patients who received DMARDs must have a period of DMARD washout that lasted for at least 4 weeks before the trial; (8) if patients took corticosteroids such as prednisone, the dose should be ≤ 10 mg, and they must have already taken more than 4 weeks before starting this study; and (9) agreed to participate in the trial and signed a form of informed consent.

Patients were excluded from this trial if they: (1) had a history of another autoimmune rheumatic disease, Sjögren's syndrome or systemic lupus erythematosus for instance; with joint swelling because of osteoarthritis, trauma, septic arthritis, or crystal arthritis; recent, current, or chronic infection, for example, the infection with hepatitis B or hepatitis C; evidence of any extents of *Mycobacterium tuberculosis* infection; (2) had other severe disorders, such as hematopoietic, brain, lung, or cardiovascular diseases; (3) had a hemoglobin level of <90 g/L, a platelet count of <100 × 10^9^/L, or a white cell count of <3.0 × 10^9^/L; (4) had an estimated glomerular filtration rate of ≤40 ml/min (evaluated by Cockcroft and Gault method); (5) with a alanine aminotransferase or aspartate aminotransferase level > 1.5 times the upper normal limit; (6) had a gastritis or active gastroduodenal ulcer induced by the long-term treatment of NSAIDs; (7) were hypersensitive to medication used in the trial; (8) had participated in any other trials within 4 weeks at the time of screening; (9) women currently pregnant or who were planning on becoming pregnant during the study period; and (10) patients with mental disease.

### Interventions

Eligible patients were allocated to receive either HQT (orally, twice per day, 250 ml for each time, 30 min after meals) or LEF (20 mg once daily) for 24 successive weeks. All the patients took MTX orally once a week, starting with 10 mg and increasing to 12.5 or 15 mg after a 4-week treatment. The ingredients and cooking method of HQT are shown in Table 1. The result observed from high-performance liquid chromatography analysis of HQT decoction is shown in Appendix 2. Patients were allowed to continue to receive NSAIDs and/or stable dosage of oral glucocorticoid (5–10 mg per day, prednisolone or equivalent) if the patients suffered intolerable pain (patient's assessment of pain ≥40 mm), folic acid, bone protection drugs such as alendronate and calcium/vitamin D, and antacids during the trial. Patients could withdraw from the trial at any time if they were not satisfied with the clinical response.

**Table 1:** Main components of HQT.

**Pinyin Name**	**Latin Name**	**Doses**
Danshen	*Salvia miltiorrhiza* Bunge	20 g
Chuanshanlong	*Dioscorea nipponica* Makino	30 g
Huangqi	*Astragalus membranaceus*	30 g
Baishao	*Paeonia tacti lora* Pall	20 g
Tianshanxuelian	*Saussurea involucrata* (Kar. et Kir.) Sch.-Bip	3 g
Duzhong	*Eucommia ulmoides* Oliver	20 g
Gusuibu	*Davallia mariesii* Moore ex Bak	20 g
Chuanxuduan	*Dipsacus asperoides* C. Y. Cheng et T. M. Ai	15 g
Shudi	Chinese Foxglove	15 g
Gancao	*Glycyrrhiza uralensis*	10 g

*HQT, Huayu-Qiangshen-Tongbi decoction. The decoction was made in the following manner: (1) Put the herbals and the right amount of cold water in the casserole, and soak the herbals in the cold water for 30 min. (2) Add 1,200 ml of cold water in the casserole, heat to boiling, and boil for 40 min, then filter the decoction two times; (3) Add 800 ml of hot water to the casserole with the boiled herbs together, boil for 30 min, and then filter the decoction two times; (4) Mix the decoctions together twice in 1 day, 250 ml for each time*.

### Outcomes and Measurements

#### Primary Outcomes

Patients were assessed concerning the outcomes and clinical parameters at baseline on weeks 4, 12, and 24 by different trained evaluators who did not know the treatments in the trial. The primary outcome was the patient's proportion achieving an ACR response of at least 20% (ACR20) at 24 weeks, according to the ACR criteria (17). To be considered as an ACR20 responder, a patient should achieve ≥20% improvement in both tender and swollen joints (28 tender and 28 swollen joints were evaluated) and ≥20% improvement in following three or more parameters: the patient's assessment of pain on a visual analog scale (0–100 mm), the physician's or patient's assessment of global health status (PaGADA/PhGADA, 0–100 mm), the patient's assessment of function with a modified version of the Health Assessment Questionnaire (HAQ, scores are based on an overall mean score ranging from the highest within each group), and the serum level of CRP or erythrocyte sedimentation (ESR).

#### Secondary Outcomes

Secondary efficacy measures were the proportion of patients with 50 or 70% improvement, ACR50 or ACR70, at week 24, the clinical disease activity index (cDAI) good response, European League Against Rheumatism (EULAR) good and moderate responses, clinical remission, and LDA. The criteria of EULAR response were evaluated based on the individual amount of change in the DAS as well as the achieved DAS (low, moderate, or high). Moderate EULAR responses were a decrease (improvement) of >0.6 and ≤1.2 and a DAS <5.1, whereas good responses are a reduction of >1.2 and a DAS <2.6 (18). A good response for cDAI was defined when achieving ≥50% improvement or a cDAI ≤2.8 (19). The extent of disease activity was assessed based on the DAS in DAS28-CRP as remission (<2.6) and LDA (<3.2) (16).

The following clinical and laboratory indexes were also assessed: the 28-joint tender joint count, 28-joint swollen joint count, morning stiffness duration, the patient's assessment of pain on a visual analog scale, PaGADA, PhGADA, CRP, ESR, rheumatoid factor (RF), HAQ score, and DAS28-CRP. Radiographs of the hands (including wrists) were performed at the screening visit and after 24-week treatment. Radiographs of the hands (including wrists) were evaluated by the van der Heijde modified total sharp score (mTSS), which was utilized to assess radiographic joint damage progression (20) taken at baseline and after 24 weeks in the trial. Sixteen and 15 areas were included for the evaluation of erosions and joint space narrowing (JSN) in hands and wrists. The maximum score of erosion was 160, and the maximum JSN score was 120. The sum of the earlier mentioned scores (maximum 280) was the mTSS. All radiographs in the trial were scored centrally in chronological order by a professional while blinded reader.

#### Safety Outcomes

Measurement of safety was evaluated by patient-expressed AEs, physical examinations, and laboratory investigations, which included a routine blood test, urine analysis, renal function, and liver function. These evaluations were undertaken at each visit during the period of treatment (baseline, 4, 12, and 24 weeks). Chest X-ray examinations and electrocardiography were conducted at the screening visit and after 24-week treatment. Hepatotoxicity was defined by the abnormal increase of the hepatic enzyme level. Hematological adverse events were assessed by the changes in hematologic characteristics, such as anemia (hemoglobin < 90 g/L), leukopenia (<3.5 × 10^9^/L), and thrombocytopenia (<100 × 10^9^/L).

### Sample Size

Due to the lack of previous similar trials and pilot studies to consult, the sample size of this pilot study was set as 45 cases in each group.

### Randomization and Blinding

An independent statistician performed randomization. SAS 9.2 software (SAS Institute Inc., Cary, USA) was used to generate the randomization sequence. Participants were randomly assigned at a 1:1 ratio by a randomization system to the MTX + HQT group or the MTX + LEF group. Blinding and placebo tables were not available for this investigator-initiated clinical trial, and the allocation sequence was not concealed from both the researchers and participants.

### Statistical Analysis

SPSS17.0 and GraphPad Prism 7 statistical software packages were used to establish the database by an independent statistician who was blinded to the group allocation. The full analysis set evaluated baseline data, and the efficacy in the two groups was assessed by both intent-to-treat (ITT) analysis and per-protocol (PP) analysis. The ITT analysis included participants who received at least 4 weeks of treatment, whereas the PP analysis only included the patients who finished 24-week treatment. The data from the patients who withdrew from the trial prematurely were considered missing, and these data were calculated using the last observation when performing the ITT analysis. Safety set analysis was used to assess the safety of two treatments, including all patients who received treatment once.

Baseline characteristics of participants were reported as the mean ± standard deviation or as numbers with corresponding percentages for categorical variables. To determine the differences in baseline characteristics between two groups, the independent *t*-tests were used for normally distributed variables, chi-square tests for categorical variables, and Mann–Whitney *U*-tests for non-normally distributed variables. Analysis of the primary endpoint (the ACR20) and some secondary efficacy endpoints (the numeration data) was analyzed by using a chi-square test or Fisher's exact test, whereas the measurement data of secondary endpoints were detected by one-way repeated measures ANOVA of the mean values from baseline to weeks 4, 12, and 24 for each group. Missing values were replaced using the last observation. All statistical tests were two-sided, which were performed at the *p* < 0.05 significance level.

## Results

### Characteristics of the Sample

**Figure 1:**
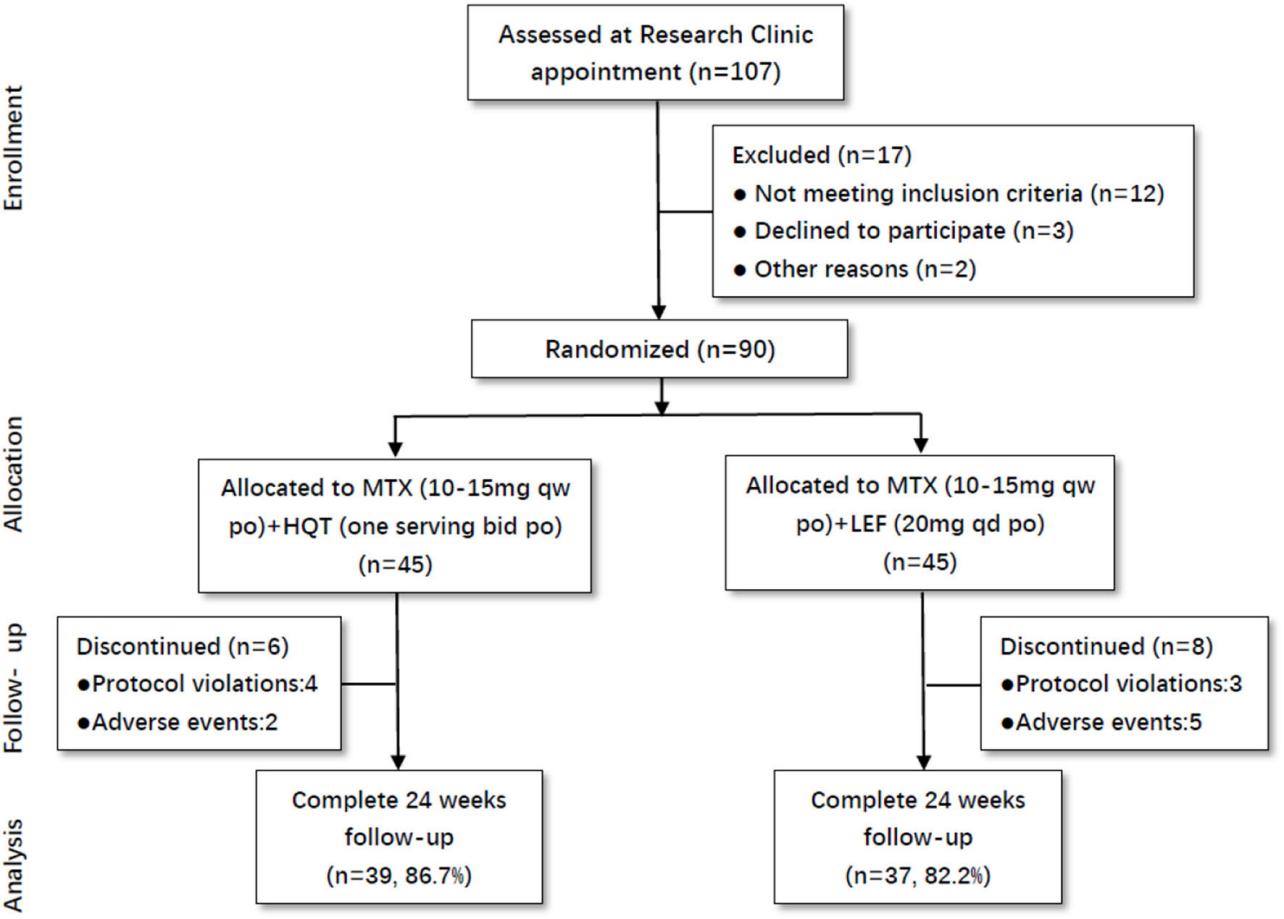
Participant flow through the trial.

Totally, 107 active RA patients were screened in this trial. Among these participants, 90 patients were eligible to be enrolled in this trial based on inclusion criteria. All of them were randomly assigned to the two groups: MTX + HQT (*n* = 45) and MTX + LEF (*n* = 45). The percentages of patients who did not finish the 24-week treatment were 13.3% in the MTX + HQT group and 17.8% in the MTX + LEF group. There were four and three patients in the MTX + HQT group and MTX + LEF group, respectively, excluded from the PP set for the protocol violation. Additionally, because of the adverse events, there were two patients in the MTX + HQT group and five patients in the MTX + LEF group who discontinued treatment (Figure 1).

There were no statistically significant differences in age, sex, demographics, or patient clinical characteristics between the two groups (*p* > 0.05). Demographics and clinical characteristics at baseline of patients with active RA are shown in Table 2. Patients received MTX at a dose of 11.84 ± 1.46 mg/week in the MTX + HQT group and 10.56 ± 1.14 mg/week in the MTX + LEF group. Concomitant medication evaluation was also performed to compare the two groups during the trial. There was no statistically significant difference in the proportion of the patients who used glucocorticoids, NSAIDs, antacids, folic acid, or calcitriol/calcium (*p* > 0.05). Besides, the mean values of the glucocorticoid doses in patients each day were 5.17 ± 0.93 mg in the MTX + HQT group and 5.0 ± 0.0 mg in the MTX + LEF group. The major concomitant medications in this study of the two groups are shown in Table 2. Furthermore, 85.6% (77/90) participants had a period of DMARD washout that lasted for at least 4 weeks before participating in this trial. Of them, 82.2% (37/45) participants were in the MTX + HQT group, and 88.9% (40/45) participants were in the MTX + LEF group; the rest of the participants, 17.8% (8/45) in the MTX + HQT group and 11.1% (5/45) in the MTX + LEF group, had never taken DMARDs. No significant difference between the two groups was observed in the rate of receiving DMARDs treatment before participating in this trial (*p* > 0.05).

**Table 2:** Demographic and characteristics data of RA patients at baseline in FAS.

**Characteristics**	**MTX + HQT (*n* = 45)**	**MTX + LEF (*n* = 45)**	***P***
Age (SD), years	51.67 (9.92)	47.56 (11.40)	0.099
Female, *n* (%)	35.00 (77.80)	31.00 (68.90)	0.340
Disease duration (SD), months	41.82 (45.93)	33.90 (36.53)	0.783
TJC (SD), *n*	8.62 (5.09)	9.20(6.15)	0.948
SJC (SD), *n*	6.29 (4.19)	4.67(3.02)	0.053
Patient's assessment of pain (SD), mm	65.09 (16.40)	59.78(20.17)	0.183
PhGADA^†^ (SD), mm	61.11 (14.81)	56.33(18.84)	0.246
PaGADA^†^ (SD), mm	62.00 (18.17)	57.56(20.47)	0.295
Morning stiffness (SD), min	47.11 (32.80)	53.89 (50.56)	0.798
HAQ, mean ± SD	0.65 (0.56)	0.92 (0.67)	0.063
hs-CRP (SD), mg/L	17.72 (19.10)	27.62 (35.15)	0.161
ESR (SD), mm/h	60.09 (27.29)	55.64 (31.39)	0.368
RF^#^ (SD), U/ml	216.15 (298.88)	180.89 (194.82)	0.812
Anti-CCP^#^, positive rate	86.05% (37/43)	87.80% (36/41)	0.811
DAS28-CRP	6.02 (1.81)	5.97 (2.17)	0.620
cDAI (SD)	27.22 (8.98)	25.12 (10.06)	0.284
**Concomitant treatments**			
NSAIDs, *n* (%)	43 (95.6%)	44 (97.8%)	1.000
Glucocorticoid oral, *n* (%)	29 (64.4%)	27 (60.0%)	0.664
Folic acid tablet, *n* (%)	41 (91.1%)	44 (97.8%)	0.357
Calcitriol/calcium carbonate, *n* (%)	37 (82.2%)	43 (95.6%)	0.094
Antacids, *n* (%)	40 (88.9%)	42 (93.3%)	0.711

*Data are presented as the mean (SD) or n (%)*.

*FAS, full analysis set; TJC, tender joint count; SJC, swollen joint count; PhGADA, physician's global assessment of disease activity; PaGADA, patient's global assessment of disease activity; HAQ, Health Assessment Questionnaire; CRP, C-reactive protein; ESR, erythrocyte sedimentation rate; RF, rheumatoid factor; anti-CCP, anti-cyclic citrullinated peptide antibody; DAS28-CRP, 28-joint disease activity score- C-reactive protein; cDAI, clinical disease activity index; NSAIDs, non-steroidal anti-inflammatory drugs*.

*RF^#^ was measured by immunonephelometric with a cutoff value of 20 U/ml. Anti-CCP^#^ was measured using a commercially available second-generation ELISA kit (Abbott, USA) with a cutoff value of 25 U/ml*.

† *Measured on a 100-mm visual analog scale; the upper limit of normal for CRP is 0–6 mg/L*.

### Clinical Efficacy

**Figure 2:**
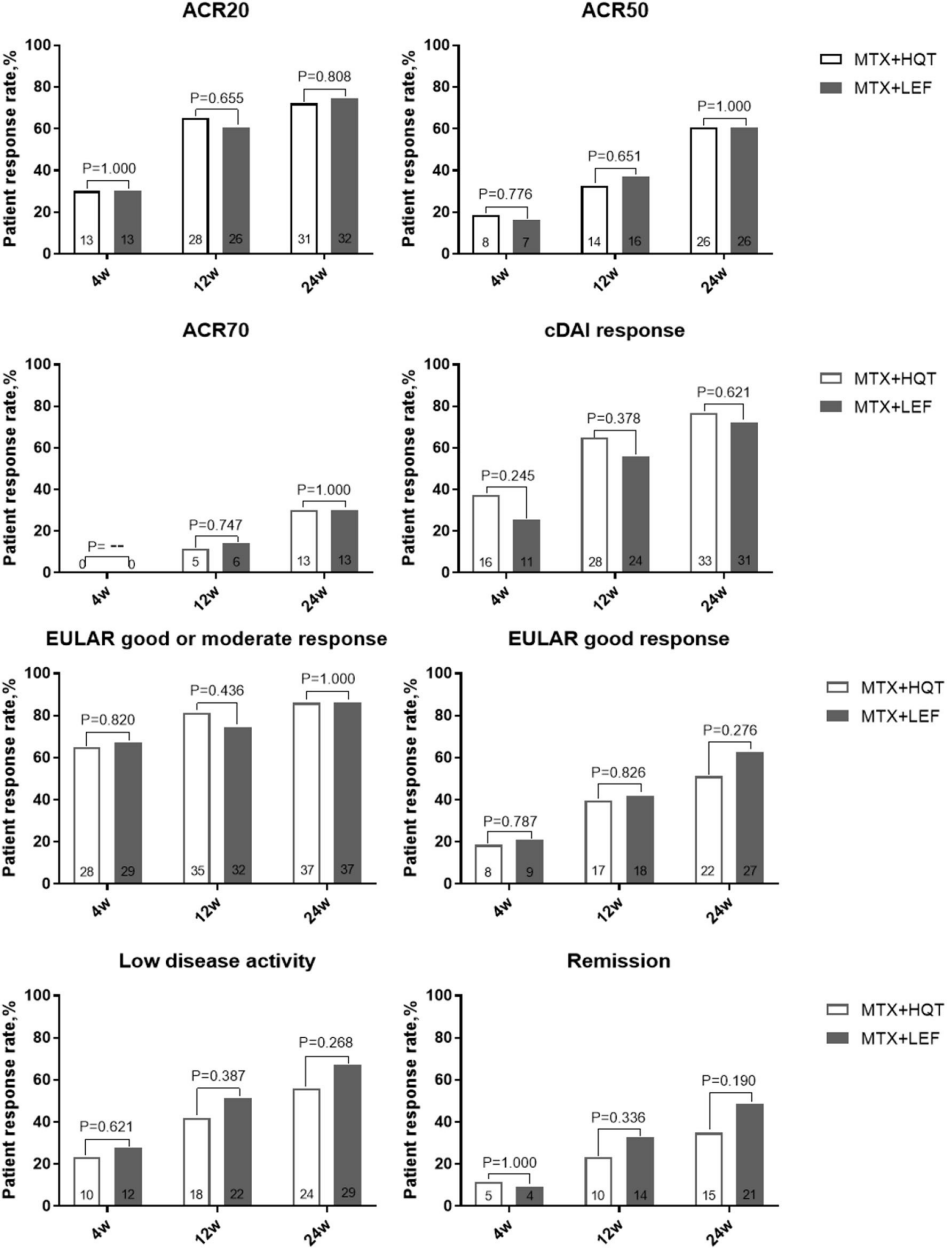
Over time measurements of primary and secondary efficacy endpoints in the ITT analysis. A comparison between two combination treatments was performed by using the chi-square test or Fisher's exact test. ACR, American College of Rheumatology; cDAI, clinical disease activity index; EULAR, European League Against Rheumatism; ITT, intention-to-treat.

In the ITT analyses after 24 weeks of treatment, there were 72.1% (31/43) and 74.4% (32/43) in the MTX + HQT and the MTX + LEF groups, respectively, who achieved the ACR20 response. Although there were more patients achieving ACR20 response in the MTX + HQT group, as compared with the MTX + LEF group, statistically, there was no difference (*p* = 0.808, Figure 2). ACR50, ACR70, cDAI good responses, EULAR good response, remission rate, and LDA rates of the patients at each evaluation point in the MTX + HQT group were similar to those in the MTX + LEF group (ACR50: 60.5 [26/43] vs. 60.5% (26/43); ACR70: 30.2 [13/43] vs. 30.2% [13/43]; cDAI good response: 76.7 [33/43] vs. 72.1% [31/43]; EULAR good or moderate response: 86.0 [37/43] vs. 86.0% [37/43]; EULAR good response: 51.2 [22/43] vs. 62.8% [27/43]; remission rate: 34.9 [15/43] vs. 48.8% [21/43]; LDA rate: 55.8 [24/43] vs. 67.4% [29/43]) (Figure 2). There was no appreciable difference between the two groups in those response rates mentioned earlier (*p* > 0.05).

In both groups, improvements in clinical symptoms (28-joint tender joint count, 28-joint swollen joint count, patient's assessment of pain, PaGADA, PhGADA, and morning stiffness duration), disease activity (DAS28-CRP), laboratory investigations (ESR, CRP, and RF), health status, and quality-of-life outcome (HAQ) were observed as early as week 4 and maintained through week 24 (*p* < 0.05). Overall, no clear differences were found between the two therapeutic regimens (*p* > 0.05). The clinical symptoms and laboratory investigations in the two groups at each point are shown in Table 3.

**Table 3:** Clinical and laboratory measures of the two groups at each visit in the ITT analysis.

**Measures**	**MTX + HQT**				**MTX + LEF**			
	**0W**	**4W**	**12W**	**24W**	**0W**	**4W**	**12W**	**24W**
TJC, *n*	8.40 (5.10)	6.47 (4.86)	4.72 (3.53)	3.56 (4.04)	8.98 (5.92)	6.36 (5.19)	4.77 (5.02)	3.32 (5.01)
SJC, *n*	6.33 (4.28)	4.05 (3.37)	2.00 (2.40)	1.81 (3.81)	4.61 (3.03)	2.91 (2.87)	1.89 (2.53)	1.07 (1.89)
Patient's assessment of pain, mm^†^	65.09 (16.75)	45.81 (17.89)	30.35 (20.66)	23.44 (18.20)	60.00 (20.35)	42.45 (22.35)	29.91 (21.30)	21.48 (19.55)
PaGADA, mm^†^	61.86 (18.55)	39.70 (20.52)	29.53 (19.75)	19.95 (17.47)	58.18 (20.26)	44.32 (22.45)	29.32 (20.95)	20.80 (20.17)
PhGADA, mm^†^	61.16 (15.15)	40.81 (20.18)	28.60 (18.85)	20.70 (16.53)	56.25 (19.05)	43.18 (20.88)	30.57 (21.41)	21.25 (19.68)
Morning stiffness, min	47.91 (33.35)	26.98 (28.10)	17.84 (32.62)	19.30 (49.07)	54.20 (51.01)	35.00 (41.81)	14.55 (18.48)	11.50 (22.49)
CRP, mg/L	18.42 (19.25)	14.36 (19.21)	12.50 (15.38)	11.52 (18.39)	27.16 (35.78)	15.07 (25.27)	18.12 (55.57)	10.13 (16.96)
ESR, mm/h	60.70 (26.44)	55.05 (27.41)	51.81 (31.30)	46.91 (27.69)	54.43 (30.79)	51.95 (32.14)	44.12 (29.49)	42.09 (28.45)
RF, U/ml	204.14 (283.21)	157.91 (222.86)	153.38 (224.95)	222.09 (437.13)	182.05 (196.06)	182.21 (246.89)	123.59 (202.98)	113.37 (149.13)
HAQ	0.64 (0.57)	0.43 (0.45)	0.38 (0.53)	0.26 (0.46)	0.91 (0.68)	0.59 (0.63)	0.44 (0.56)	0.31 (0.57)
DAS28-CRP	5.98 (1.85)	4.73 (2.08)	3.69 (1.60)	3.21 (1.77)	5.90 (2.09)	4.47 (2.04)	3.64 (2.09)	2.98 (2.05)

* *Values are the mean (SD)*.

*TJC, tender joint count; SJC, swollen joint count; PaGADA, patient's global assessment of disease activity; PhGADA, physician's global assessment of disease activity; HAQ, Health Assessment Questionnaire; ESR, erythrocyte sedimentation rate; CRP, C-reactive protein; RF, rheumatoid factor; DAS28, 28-joint disease activity score*.

† *Measured on a 100-mm visual analog scale*.

**Figure 3:**
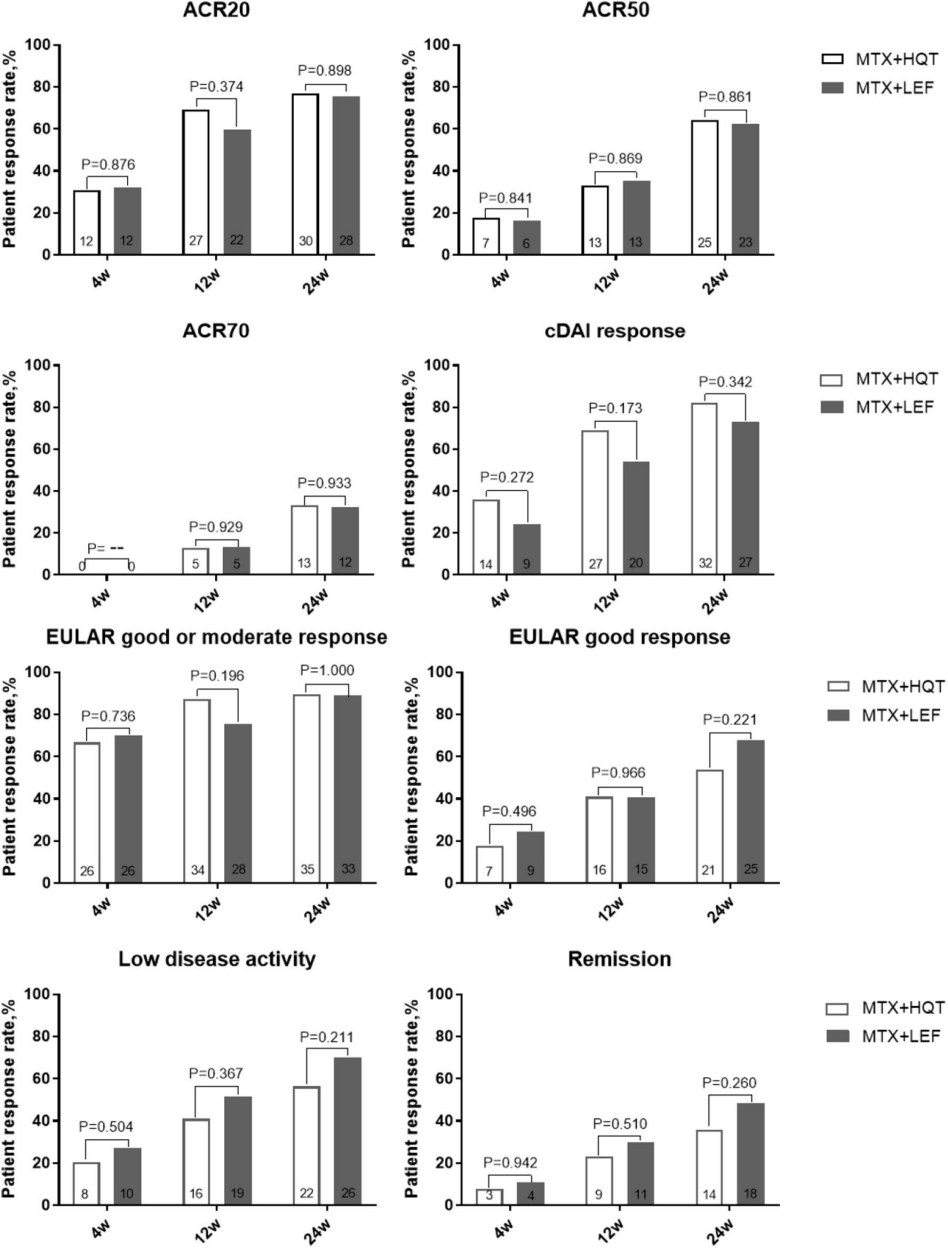
Over time measurements of primary and secondary efficacy endpoints in the PPS analysis. A comparison between two combination treatments was performed by using the chi-square test or Fisher's exact test. ACR, American College of Rheumatology; cDAI, clinical disease activity index; EULAR, European League Against Rheumatism; PPS, per-protocol set.

Additionally, we performed a PP analysis of the data from the patients who finished the 24-week treatment. At 24 weeks, ACR20 responses were attained in 76.9% (30/39) patients who received MTX and HQT and 75.7% (28/37) patients who received MTX and LEF, and no statistical significance was observed between the two groups (*p* > 0.05). The result of the PP analysis was in agreement with those found in the ITT analysis. Similar results of statistical analyses were seen for ACR50, ACR70, EULAR good response, cDAI good response, clinical remission, and LDA rate at week 24 in the PP analysis (Figure 3). A full list of the mean (standard deviation) on clinical symptoms and laboratory investigations at each point is provided in Appendix 3. After treatment, the clinical symptoms, laboratory investigations, HAQ score, and DAS were significantly improved compared with those before (*p* < 0.05). No statistical significance was observed in the improvement of those measures from baseline to week 24 between the two groups (*p* > 0.05).

### Evaluation of the Radiographic Joint Damage

After 24-week treatment, 82.2% (37/45) of the patients in the MTX + HQT group provide the reports of radiographs at the two evaluation points and 71.1% (32/45) of the patients in the MTX + LEF group. Mean change values from baseline at week 24 were 3.59 ± 4.75 and 1.34 ± 8.67 with mTSS, 1.24 ± 2.39 and 0.63 ± 3.78 with JSN scores, 2.35 ± 2.96 and 0.72 ± 5.48 with erosions scores in the MTX + HQT and MTX + LEF groups, respectively; no significant differences were found between the two groups (*p* > 0.05). Comparing with the baseline, mTSS and erosions scores resulted in significant differences in the patients treated with two different treatment regimens at week 24 (*p* < 0.05), so as the JSN score in the MTX + HQT group (*p* < 0.05), whereas no significant differences of JSN score were observed in the MTX + LEF group (*p* > 0.05). Radiographs of the hands (including wrists) assessed by mTSS are shown in Table 4.

**Table 4:** Mean (SD) in the van der Heijde modified total sharp score (mTSS) in the two groups.

**Joint damage**	**MTX + HQT (*N* = 37)**	**MTX + LEF (*N* = 32)**	***P* (between groups)**
**mTSS**			
Baseline, mean (SD)	50.92 (53.66)	36.44 (32.46)	0.736
Week 24, mean (SD)	54.51 (55.80)	37.78 (31.70)	0.613
*P* value (within the group)	<0.01	0.013	
**JSN SCORE**			
Baseline, mean (SD)	23.92 (25.46)	15.47 (14.87)	0.535
Week 24, mean (SD)	25.16 (26.33)	16.09 (14.35)	0.413
*P* value (within the group)	0.002	0.132	
**EROSION SCORE**			
Baseline, mean (SD)	27.00 (30.33)	20.97 (19.53)	0.928
Week 24, mean (SD)	29.35 (31.60)	21.69 (19.35)	0.814
*P*-value (within the group)	<0.01	0.017	

*mTSS, the van der Heijde modified total sharp score; JSN, joint space narrowing; SD, standard deviation*.

### Safety and Tolerability

Safety evaluation was performed in the safety set analysis; all adverse events reported in this trial are listed in Table 5. In total, 28 patients (31.1%) experienced one or more adverse events (11 cases in the MTX + HQT group and 17 cases in the MTX + LEF group). The most common adverse events related to MTX + HQT were gastrointestinal discomfort, and all of the participants alleviated quickly and continued our trial after treatment with an antacid, although hepatic dysfunction, and gastrointestinal discomfort were the most common adverse events with MTX + LEF, and five patients withdrew from the trial (four patients for hepatic dysfunction and one for hypertension). There was no statistical significance between the two groups in the incidences of all adverse events (*p* > 0.05).

**Table 5:** Summary of adverse events in the safety analysis set.

**Adverse events**	**MTX + HQT (*n* = 45)**	**MTX + LEF (*n* = 45)**	***P***
All	11 (24.4%)	17 (37.8%)	
ALT/AST elevation	0 (0%)	4 (8.9%)	0.117
Gastrointestinal reactions	9 (20.0%)	10 (22.2%)	0.796
Rash	1 (2.2%)	1 (2.2%)	1.000
Atrial fibrillation	1 (2.2%)	0 (0%)	1.000
Leukopenia	0 (0%)	1 (2.2%)	1.000
Hypertension	0 (0%)	1 (2.2%)	1.000

*Data are presented as n (%)*.

*ALT, alanine aminotransferase; AST, aspartate aminotransferase*.

## Discussion

Over thousands of years, TCM has been beneficial to many patients in China. Nowadays, TCM is regarded as a basic or complementary therapy for RA patients, and, therefore, a variety of TCM herbs have been used in the clinic for RA treatment. The anti-inflammatory and antiarthritic activities of many TCM herbs have been validated in arthritic models and also tested in clinical trials in patients with RA (10, 19, 21). Thus, Chinese herbals may also function as DMARDs, which could be used as an alternative or basic treatment for RA patients.

In TCM theory, RA belongs to “Bi” disease, which is a group of disorders with symptoms and signs similar to arthritis or other rheumatism defined in Western medicine (22–24). The development of “Bi” syndrome is due to an evil spirit, the pathogeny of TCM, including wind, cold, and wet that invade the human body and lead to poor circulation of Qi and blood, so-called “blood stasis” (23, 25, 26). Based on years of clinical experience and observation, “removing blood stasis” theory of TCM and the use of blood-activating herbs had efficacy in relieving clinical symptoms, signs, and indicators of inflammatory activity in RA patients (27). HQT is a Chinese herbal formula, and it is prepared for treating RA by activating blood circulation, dissipating blood stasis, and dispelling pathogenic wind, cold, and wet.

During the 24-week trial, the treatment with MTX + HQT resulted in significant improvement in clinical signs and symptoms of RA, including joint pain, joint swelling, morning stiffness duration, and measures of quality-of-life outcome, as well as in many inflammatory indicators, such as CRP, ESR, and the autoantibody RF. Comparing with MTX + LEF (a recommended therapy for refractory RA) (28–30), MTX + HQT led to a similar improvement in terms of patients achieving ACR20, ACR50, ACR70, cDAI, LDA, and remission responses and to moderate or good improvement in DAS28-CRP. In this pilot study, the combinational therapy of HQT with MTX effectively and safely alleviated symptoms and signs of patients with active RA. It is well-known that the destruction of smaller joints more frequently attacks the RA patients and that radiographs of both hands and feet are the most popular standard to evaluate structural changes, which is regarded as one of the criteria for assessing therapeutic efficacy (31). After 24-week treatment, both groups had higher mTSS than before treatment. However, X-ray analyses in our study showed no statistical difference in terms of mTSS between the two groups in the progression of radiographic joint damage. We considered that increasing mTSS might be in association with the high proportion (60.9%) of patients with disease duration of 2 years or more who enrolled in radiographic analyses set.

Our trial showed for the first time that MTX combined with Chinese herbal formula is equivalently effective as MTX combined with LEF in active RA patients. Previous studies have shown that herbal medicine monotherapy or combination therapy has efficacy in relieving clinical symptoms, signs, and indicators of inflammatory activity for RA patients (12, 32). According to previous pharmacological studies, the herbals in HQT formula are proven to have a variety of pharmacological effects, such as anti-inflammatory properties, analgesia, and immune suppression (33, 34), supporting the clinical efficacy of HQT in RA treatment. The root and rhizoma of *S. miltiorrhiza* Bunge (Danshen) and the rhizoma of *D. nipponica* Makino (Chuanshanlong) are the most important components of HQT. Previous pharmacological studies have demonstrated that *S. miltiorrhiza* injection could inhibit the proliferation of fibroblast-like synoviocytes obtained from RA patients (35, 36). Also, tanshinone VI, an abietane diterpene extracted from the root of *S. miltiorrhiza* Bunge, could improve bone loss by inhibiting osteoclastic bone resorption through inhibition of nuclear factor-κB and receptor activator of nuclear factor kappa-κ ligand pathways (37). Diosgenin, a major alkaloid monomer from the rhizoma of *D. nipponica* Makino, has a variety of pharmacological effects to relieve pain, reduce inflammation, regulate cytokine expression, and inhibit the proliferation of fibroblast-like synoviocytes, and, therefore, it has been frequently used to treat RA (38, 39). Many pharmacological studies reported that *D. mariesii* Moore ex Bak (Gusuibu), *D. asperoides* C. Y. Cheng et T. M. Ai (Chuanxuduan), and *E. ulmoides* Oliver (Duzhong) hold the potentials to prevent osteoporosis and inflammation associated with arthritis (40–42). Our previous study demonstrated that the combined therapy of HQT and MTX could significantly improve the clinical symptoms of RA patients with good tolerance (13). Furthermore, many experimental studies showed that the active ingredients of the other six herbs in HQT also exert anti-arthritic effects in both *in vivo* and *in vitro* models (43–47). Pieces of evidence from these pharmacological or mechanism studies support the clinical efficacy of MTX + HQT observed in our trial.

In our current study, all AEs were predominantly mild or moderate, with a low incidence rate. Gastrointestinal reactions and liver abnormalities were AEs. Reported AEs suggested that the most significant safety issue of MTX + LEF combination was potential liver toxicity, which was undoubtedly consistent with the real situation of LEF usage in RA treatment (48). In our study, liver abnormalities occurred in 8.9% of the MTX + LEF, which was lower than those reported in several other RCTs in RA (28, 29). The result of the previous study found that the incidence of alanine aminotransferase/aspartate aminotransferase elevations increased ~ 2–5 folds in the combination of MTX and LEF, which depended on the dosages of MTX (48). In our study, we used the low dose of MTX (10–12.5 mg/week), reflecting the standard of the Chinses recommendations for the management of RA currently, which might be one of the reasons for the low rate of adverse events that occurred in our trial. Besides, more than 90% of patients in our trial received folic acid, which had the efficacy of lessening toxicity without altering efficacy during long-term treatment with MTX for RA (49).

Several critical factors in the course of analyzing the observational data should be considered in this work. Firstly, this trial was an open-label, monocenter, clinical trial, and the treating physicians and patients were not blinded to medication. To make an objective assessment of the efficacy and safety of the MTX + HQT combination therapy, the clinical outcomes were assessed and analyzed by evaluators and statisticians who were unaware of the therapy. However, a completely objective assessment needs to be verified in a multicenter, double-blind RCT in the future. Secondly, it was a 24-week observation trial, which may not be sufficient to show the long-term benefit of the MTX + HQT combination therapy, especially the radiographic progression. Thirdly, this trial did not compare the HQT in monotherapy with the MTX or another csDMARD in monotherapy, so the clinical efficacy of HQT itself cannot be evaluated or compared directly. Finally, due to a lack of similar clinical studies and pilot studies to reference, the sample size and the hypothesis test type (non-inferiority trial, equivalence trial, or superiority trial) in the design of this pilot study could not be pre-estimated accurately. However, the result of this pilot study can be the basic data and reference for further study.

## Conclusion

This pilot study was the first time to evaluate the effect and safety of HQT, a Chinese medicine formula, combined with MTX, comparing with the combination of MTX and LEF. The results of this analysis indicate that the therapeutic regimen of HQT combined with MTX provides a potentially beneficial clinical response with acceptable tolerability for treating patients with active RA, which implies that HQT or other Chinese medicine formula may be a good therapeutic option in combination with MTX for RA treatment. However, it should be stressed that interpretations of the efficacy data are limited by the shortcoming mentioned earlier. A multicentric, double-blinded, preferably placebo-controlled, as well as with a longer follow-up, RCT is motivated to definitively establish the efficacy and safety of the HQT + MTX combination therapy and even the HQT in monotherapy.

## Data Availability Statement

The datasets generated for this study are available on request to the corresponding author.

## Ethics Statement

The studies involving human participants were reviewed and approved by The Medical Ethics Committee of the Second Affiliated Hospital of Guangzhou University of Chinese Medicine (B2016-076-01). The patients/participants provided their written informed consent to participate in this study.

## Author Contributions

XiaC, YL, YZ, XiuC, YC, XW, ZW, P-JJ, QH, and RH: design and conception. JW, KG, AO, ZL, YL, YZ, and XiuC: acquisition, analysis, and interpretation. JZ, JP, ZH, XiuC, JW, KG, QH, and RH: major contributors in writing the manuscript. All authors read and approved the final manuscript. All authors contributed to the study design and review before submission according to their interests and scientific expertise.

## Conflict of Interest

The authors declare that the research was conducted in the absence of any commercial or financial relationships that could be construed as a potential conflict of interest.

## Abbreviations

ACR: American College of Rheumatology criteria
ACR: American College of Rheumatology
ANOVA: analysis of variance
cDAIs: clinical disease activity index good responses
csDMARDs: conventional synthetic DMARDs
DAS28-CRP: 28-joint disease activity score based on C-reactive protein
DMARDs: disease-modifying antirheumatic drugs
EULAR: European League against rheumatism
ESR: erythrocyte sedimentation
HQT: Huayu-Qiangshen-Tongbi decoction
HAQ: Health Assessment Questionnaire
ITT: intention-to-treat
JSN: joint space narrowing
LDA: low disease activity
LEF: Leflunomide
mTSS: van der Heijde modified total sharp score
MTX: methotrexate
NSAIDs: Nonsteroidal anti-inflammatory drugs
PaGADA: physician's or patient's assessment of global health status
PhGADA: patient's assessment of global health status
PP: per-protocol
RA: rheumatoid arthritis
RCT: randomized controlled trial
RF: rheumatoid factor
TB: tuberculosis
TCM: Traditional Chinese medicine.

## References

[1] Mcinnes IB Schett G. The pathogenesis of rheumatoid arthritis. N Engl J Med. (2011) 365:2205–19. 10.1056/NEJMra1004965 22150039

[2] Sepriano A Kerschbaumer A Smolen JS Van Der Heijde D Dougados M Van Vollenhoven R Safety of synthetic and biological DMARDs: a systematic literature review informing the 2019 update of the EULAR recommendations for the management of rheumatoid arthritis. Ann Rheum Dis. (2020) 7:760–70. 10.1136/annrheumdis-2019-216653 32033941

[3] Chatzidionysiou K Sfikakis PP. Low rates of remission with methotrexate monotherapy in rheumatoid arthritis: review of randomized controlled trials could point towards a paradigm shift. RMD Open. (2019) 5:e000993. 10.1136/rmdopen-2019-000993 31413870 PMC6667970

[4] Badavanis G Pasmatzi E Monastirli A Tsambaos D. Biologic agents in systemic dermatotherapy: cutaneous and systemic side effects. Curr Drug Saf . (2017) 12:76–94. 10.2174/1574886312666170518124014 28521707

[5] Jick SS Lieberman ES Rahman MU Choi HK. Glucocorticoid use, other associated factors, ad the risk of tuberculosis. Arthritis Rheum. (2006) 55:19–26. 10.1002/art.21705 16463407

[6] Novosad SA Winthrop KL. Beyond tumor necrosis factor inhibition: the expanding pipeline of biologic therapies for inflammatory diseases and their associated infectious sequelae. Clin Infect Dis. (2014) 58:1587–98. 10.1093/cid/ciu104 24585557

[7] Du Pan SM Dehler S Ciurea A Ziswiler HR Gabay C Finckh A. Comparison of drug retention rates and causes of drug discontinuation between anti-tumor necrosis factor agents in rheumatoid arthritis. Arthritis Rheum. (2009) 61:560–8. 10.1002/art.24463 19405000

[8] Cohen SB Czeloth N Lee E Klimiuk PA Peter N. Long-term safety, efficacy, and immunogenicity of adalimumab biosimilar BI 695501 and adalimumab reference product in patients with moderately-to-severely active rheumatoid arthritis: results from a phase 3b extension study (VOLTAIRE-RAext). Expert Opin Biol Ther. (2019) 19:1097–105. 10.1080/14712598.2019.1645114 31387417

[9] Lu MC Livneh H Chiu LM Lai NS Yeh CC Tsai TY. A survey of traditional Chinese medicine use among rheumatoid arthritis patients: a claims data-based cohort study. Clin Rheumatol. (2019) 38:1393–400. 10.1007/s10067-018-04425-w 30671749

[10] Huang RY Pan HD Wu JQ Zhou H Li ZG Qiu P Comparison of combination therapy with methotrexate and sinomenine or leflunomide for active rheumatoid arthritis: A randomized controlled clinical trial. Phytomedicine. (2019) 57:403–10. 10.1016/j.phymed.2018.12.030 30851515

[11] Chen XM Wu JQ Huang QC Zhang JY Pen JH Huang ZS. Systematic review and meta-analysis of the efficacy and safety of Biqi capsule in rheumatoid patients arthritis. Exp Ther Med. (2018) 15:5221–30. 10.3892/etm.2018.6121 29904406 PMC5996666

[12] He YT Ou AH Yang XB Chen W Fu LY Lu AP. Traditional Chinese medicine versus western medicine as used in China in the management of rheumatoid arthritis: a randomized, single-blind, 24-week study. Rheumatol Int. (2014) 34:1647–55. 10.1007/s00296-014-3010-6 24760484

[13] Lv Y Chen XH Huang RY Zhao Y Wu JQ Chen XM Clinical analysis of chinese medicine compound huayu-qiangshen-tongbi decoction combined with Methotrexate for the treatment of chinese patients with rheumatoid arthritis: a retrospective study. Zhongguo Zhong Xi Yi Jie He Za Zhi. (2019) 39:547–52.

[14] Arnett FC Edworthy SM Bloch DA Mcshane DJ Fries JF Cooper NS. The American rheumatism association 1987 revised criteria for the classification of rheumatoid arthritis. Arthritis Rheum. (1988) 31:315–24. 10.1002/art.1780310302 3358796

[15] Aletaha D Neogi T Silman AJ Funovits J Felson DT Bingham CO. 2010 Rheumatoid arthritis classification criteria: an American College of Rheumatology/European League Against Rheumatism collaborative initiative. Arthritis Rheum. (2010) 62:2569–81. 10.1002/art.27584 20872595

[16] Madsen OR. Is DAS28-CRP with three and four variables interchangeable in individual patients selected for biological treatment in daily clinical practice? Clin Rheumatol. (2011) 30:1577–82. 10.1007/s10067-011-1847-6 21956233

[17] Felson DT Anderson JJ Boers M Bombardier C Furst D Goldsmith C. American college of rheumatology. Preliminary definition of improvement in rheumatoid arthritis. Arthritis Rheum. (1995) 38:727–35. 10.1002/art.1780380602 7779114

[18] Fransen J Van Riel PL. The disease activity score and the EULAR response criteria. Rheum Dis Clin North Am. (2009) 35:745–57. 10.1016/j.rdc.2009.10.001 19962619

[19] Lv QW Zhang W Shi Q Zheng WJ Li X Chen H Comparison of *Tripterygium wilfordii* hook F with methotrexate in the treatment of active rheumatoid arthritis (TRIFRA): a randomised, controlled clinical trial. Ann Rheum Dis. (2015) 74:1078–86. 10.1136/annrheumdis-2013-204807 24733191

[20] Van Der Heijde D How to read radiographs according to the Sharp/van der Heijde method. J Rheumatol. (2000) 27:261–3. 10.1097/00002281-200007000-00005 10648051

[21] Venkatesha SH Rajaiah R Berman BM Moudgil KD. Immunomodulation of autoimmune arthritis by herbal CAM. Evid Based Complement Alternat Med. (2011) 2011:986797. 10.1155/2011/986797 21234398 PMC3014691

[22] Zhang C Jiang M Lu AP Evidence-based Chinese medicine for rheumatoid arthritis. J Tradit Chin Med. (2011) 31:152–7. 10.1016/S0254-6272(11)60031-9 21977818

[23] Lo LC Chen CY Chiang JY Cheng TL Lin HJ Chang HH. Tongue diagnosis of traditional Chinese medicine for rheumatoid arthritis. Afr J Tradit Complement Altern Med. (2013) 10:360–9. 10.4314/ajtcam.v10i5.24 24311851 PMC3847431

[24] Wang M Chen G Lu C Xiao C Li L Niu X. Rheumatoid arthritis with deficiency pattern in traditional Chinese medicine shows correlation with cold and hot patterns in gene expression profiles. Evid Based Complement Alternat Med. (2013) 2013:248650. 10.1155/2013/248650 24174973 PMC3794642

[25] Zhou X Zhou Z Jin M Wang H Wu M Gu Q. Intermediate and late rheumatoid arthritis treated by tonifying the kidney, resolving phlegm and removing blood stasis. J Tradit Chin Med. (2000) 20:87–92. 11038992

[26] Chu YL Jiang YQ Sun SL Zheng BL Xiong WS Li WJ. The differential profiles of long non-coding RNAs between rheumatoid arthritis and gouty arthritis. Discov Med. (2017) 24:133–46. 29272690

[27] Huang QC Chu YL He XH Huang RY. Regulatory roles of compound danshen in the downstream path of cyclooxygenases in rheumatoid arthritis patients' synovium. Zhongguo Zhong Xi Yi Jie He Za Zhi. (2013) 33:1416–9. 24432692

[28] Antony T Jose VM Paul BJ Thomas T. Efficacy and safety of leflunomide alone and in combination with methotrexate in the treatment of refractory rheumatoid arthritis. Indian J Med Sci. (2006) 60:318–26. 10.4103/0019-5359.26608 16864918

[29] Londono J Santos AM Santos PI Cubidez MF Guzman C Valle-Onate R. Therapeutic efficacy and safety of methotrexate + leflunomide in Colombian patients with active rheumatoid arthritis refractory to conventional treatment. Rev Bras Reumatol. (2012) 52:837–45. 10.1590/S0482-50042012000600003 23223695

[30] Hodkinson B Magomero KR Tikly M. Combination leflunomide and methotrexate in refractory rheumatoid arthritis: a biologic sparing approach. Ther Adv Musculoskelet Dis. (2016) 8:172–9. 10.1177/1759720X16664324 27721903 PMC5037498

[31] Fransen J Van Riel PL. Outcome measures in inflammatory rheumatic diseases. Arthritis Res Ther. (2009) 11:244. 10.1186/ar2745 19849821 PMC2787283

[32] Lee S Cho Y Kim J Kang JW Yoon GY Lee JH. The efficacy and safety of the herbal medicine geonchildan for patients with active rheumatoid arthritis: study protocol for a randomized, double-blind, placebo-controlled, parallel pilot trial. Trials. (2018) 19:471. 10.1186/s13063-018-2849-3 30176923 PMC6122614

[33] Wang Z Lin HH Linghu K Huang RY Li G Zuo H. Novel compound-target interactions prediction for the herbal formula hua-yu-qiang-shen-tong-bi-fang. Chem Pharm Bull. (2019) 67:778–85. 10.1248/cpb.c18-00808 31366827

[34] Wang Z Linghu KG Hu Y Zuo H Yi H Xiong SH. Deciphering the pharmacological mechanisms of the huayu-qiangshen-tongbi formula through integrating network pharmacology and *in vitro* pharmacological investigation. Front Pharmacol. (2019) 10:1065. 10.3389/fphar.2019.01065 31607918 PMC6767993

[35] Liu QS Zhu XC Li JA Xing Y Jiang H Zhang J. Effects of danshen injection on the proliferation of rheumatoid arthritis fibroblast-like synoviocytes cultured with human serum. Zhongguo Zhong Xi Yi Jie He Za Zhi. (2013) 33:674–8. 23905390

[36] Jie L Du H Huang Q Wei S Huang R Sun W. Tanshinone IIA induces apoptosis in fibroblast-like synoviocytes in rheumatoid arthritis via blockade of the cell cycle in the G2/M phase and a mitochondrial pathway. Biol Pharm Bull. (2014) 37:1366–72. 10.1248/bpb.b14-00301 24920239

[37] Nicolin V Dal Piaz F Nori SL Narducci P De Tommasi N. Inhibition of bone resorption by Tanshinone VI isolated from Salvia miltiorrhiza Bunge. Eur J Histochem. (2010) 54:e21. 10.4081/ejh.2010.e21 20558342 PMC3167308

[38] Liagre B Vergne-Salle P Corbiere C Charissoux JL Beneytout JL. Diosgenin, a plant steroid, induces apoptosis in human rheumatoid arthritis synoviocytes with cyclooxygenase-2 overexpression. Arthritis Res Ther. (2004) 6:R373–83. 10.1186/ar1199 15225373 PMC464911

[39] Liagre B Leger DY Vergne-Salle P Beneytout JL. MAP kinase subtypes and Akt regulate diosgenin-induced apoptosis of rheumatoid synovial cells in association with COX-2 expression and prostanoid production. Int J Mol Med. (2007) 19:113–22. 10.3892/ijmm.19.1.113 17143555

[40] Jung HW Jung JK Son KH Lee DH Kang TM Kim YS. Inhibitory effects of the root extract of Dipsacus asperoides C.Y. Cheng et al. T.M.Ai on collagen-induced arthritis in mice. J Ethnopharmacol. (2012) 139:98–103. 10.1016/j.jep.2011.10.020 22041103

[41] Lin TH Yang RS Wang KC Lu DH Liou HC Ma Y. Ethanol extracts of fresh *Davallia formosana* (WL1101) inhibit osteoclast differentiation by suppressing RANKL-induced nuclear factorkappa B activation. Evid Based Complement Alternat Med. (2013) 2013:647189. 10.1155/2013/647189 24191169 PMC3804452

[42] Wang JY Yuan Y Chen XJ Fu SG Zhang L Hong YL. Extract from Eucommia ulmoides Oliv. ameliorates arthritis via regulation of inflammation, synoviocyte proliferation and osteoclastogenesis in vitro and in vivo. J Ethnopharmacol. (2016) 194:609–16. 10.1016/j.jep.2016.10.038 27743778

[43] Jiang JB Qiu JD Yang LH He JP Smith GW Li HQ. Therapeutic effects of astragalus polysaccharides on inflammation and synovial apoptosis in rats with adjuvant-induced arthritis. Int J Rheum Dis. (2010) 13:396–405. 10.1111/j.1756-185X.2010.01555.x 21199477

[44] Zhou J Xu G Yan J Li K Bai Z Cheng W. Rehmannia glutinosa (Gaertn.) DC polysaccharide ameliorates hyperglycemia, hyperlipemia and vascular inflammation in streptozotocin-induced diabetic mice. J Ethnopharmacol. (2015) 164:229–38. 10.1016/j.jep.2015.02.026 25698243

[45] Xu M Guo Q Wang S Wang N Wei L Wang J. Anti-rheumatoid arthritic effects of *Saussurea involucrata* on type II collagen-induced arthritis in rats. Food Funct. (2016) 7:763–70. 10.1039/C5FO00603A 26508519

[46] Luo J Song WJ Xu Y Chen GY Hu Q Tao QW. Benefits and safety of tripterygium glycosides and total glucosides of paeony for rheumatoid arthritis: an overview of systematic reviews. Chin J Integr Med. (2019) 25:696–703. 10.1007/s11655-019-3221-5 31385219

[47] Zhai KF Duan H Cui CY Cao YY Si JL Yang HJ. Liquiritin from glycyrrhiza uralensis attenuating rheumatoid arthritis via reducing inflammation, suppressing angiogenesis, and inhibiting MAPK signaling pathway. J Agric Food Chem. (2019) 67:2856–64. 10.1021/acs.jafc.9b00185 30785275

[48] Curtis JR Beukelman T Onofrei A Cassell S Greenberg JD Kavanaugh A Elevated liver enzyme tests among patients with rheumatoid arthritis or psoriatic arthritis treated with methotrexate and/or leflunomide. Ann Rheum Dis. (2010) 69:43–7. 10.1136/ard.2008.101378 19147616 PMC2794929

[49] Shea B Swinden MV Ghogomu ET Ortiz Z Katchamart W Rader T. Folic acid and folinic acid for reducing side effects in patients receiving methotrexate for rheumatoid arthritis. J Rheumatol. (2014) 41:1049–60. 10.3899/jrheum.130738 24737913

