# Supplementary material for: Chinese Herbal Formula Huayu-Qiangshen-Tongbi Decoction Compared With Leflunomide in Combination With Methotrexate in Patients With Active Rheumatoid Arthritis: An Open-Label, Randomized, Controlled, Pilot Study
Source: Front Med (Lausanne). 2020 Sep 4;7:484. doi: 10.3389/fmed.2020.00484 (PMC7498571; doi:10.3389/fmed.2020.00484)
Supplement: Supplementary file 1 [file Table_1.DOCX]

**Appendix 1**

**Table of Patient’s Clinical Information of TCM**

Patient’s name: Gender: □male □female

Date: Physician’s name:

**1.Observation of the tongue：**

**2.Feeling of the pulse:**

**3.TCM syndromes：**

□Wind cold and wet resistance

□Rheumatic heat depression

□phlegm and static blood binding together

□Kidney deficiency and cold coagulation

□Yin deficiency of liver and kidney

□Deficiency of Qi and blood

**4. Figure 1. TCM symptom grading scale**

| Symptoms | Standard for Evaluation | | | | Score |
| --- | --- | --- | --- | --- | --- |
|  | **0** | **1** | **3** | **5** |  |
| Range of motion of the fingers and wrists | Normal | Joint activity was slightly restricted and range of motion was reduced by less than 1/3. | Joint activity was significantly restricted and the range of motion decreased by 1/3 to 2/3. | Joint activity was obviously restricted and the range of motion decreased by more than 2/3 or even rigidity. |  |
| The temperature of joints (Hot) | Normal | The patient didn’t feel hot in his/her joints, but the temperature of the joints is hotter than others in physical examination. | The patient felt slightly hot in his/her joints, and the temperature of the joints is hotter than others in physical examination. | The patient felt scorching in his/her joints, and the temperature of the joints is much hotter than others in physical examination. |  |
| The temperature of joints (Cold) | Normal | The patient felt cold in his/her joints, but the temperature of the joints is normal in physical examination. | The patient felt cold in his/her joints, and the temperature of the joints is colder than others in physical examination. | The patient felt seriously cold in his/her joints, and the temperature of the joints is colder than others in physical examination. |  |
| Thirsty | Never | Sometimes | Thirty and drink water in moderation | Thirty and drink a lot of water |  |
| Sweating | Rare | Sometimes | Sweating easily when take exercise | Sweating easily when stay in silence |  |
| [Aversion to wind and cold](http://www.baidu.com/link?url=ibJrYhBZhJXDO5qozWNzeEurmeXwPb0qVcJ_eTnGX8OBC3GuiH20D4_wuOYQlkMEnN7NrV_EpESTpLPN4baifyiuo9oaKzNOO0whcX6L_cDM91p0cTOknXNqr1QX_c30eNs8Lc35R0l0lPD3V8vM3q) | Never | Sometimes | Often, no need to wear more clothes | Often, relieve by wearing more clothes |  |
| Limb cold | Never | The patient felt cold in his/her limbs, but the temperature of the limbs is normal in physical examination. | The patient felt cold in his/her limbs, and the temperature of the limbs is lower than skin temperature nearby in physical examination. | The patient felt cold and heavy in his/her limbs, and has obvious pain. |  |
| [Soreness and weakness of waist and knees](http://www.baidu.com/link?url=1ZGyVwLzGoe-NE1avHs5B5gzAXwAOU8Ck40vptHuZN3g_l6xVkRCQI4_uwc3-NCl1KNdMN2mMjoh5F_uphOX6vPb2djHvmGSpw8LUwBVyzVpW1LG1bAuLAtMIwoK_nH5QdfJe7USHCjCvGGCk7I2aKHsa-3GnkEf3WTNWOkQu2i) | Never | Sometimes | Often | All the time |  |
| Morning stiffness | Never | Time of morning stiffness less than 1 hour | Time of morning stiffness between 1 to 2 hours | Time of morning stiffness more than 2 hours |  |

| Symptom of Joints | |
| --- | --- |
| Pain | □crymodynia □Prick □causalgia □Aching pain □heavy □scorching |
| Position | □steadfast □[peripatetic](http://dict.youdao.com/w/peripatetic/#keyfrom=E2Ctranslation) |
| Aggravation or Mitigation | □The symptoms will aggravate in cold condition, and relieve in warm condition.  □The symptoms will aggravate in warm condition, and relieve in cold condition.  □The symptoms will aggravate in the day, and relieve in the night.  □The symptoms will aggravate in the night, and relieve in the day. |
| Characteristic of Joints | □Swollen □Joints activity were slightly restricted □Stiffness □Joints activity were obviously restricted □malformation |
