# Supplementary material for: Chinese Herbal Formula Huayu-Qiangshen-Tongbi Decoction Compared With Leflunomide in Combination With Methotrexate in Patients With Active Rheumatoid Arthritis: An Open-Label, Randomized, Controlled, Pilot Study
Source: Front Med (Lausanne). 2020 Sep 4;7:484. doi: 10.3389/fmed.2020.00484 (PMC7498571; doi:10.3389/fmed.2020.00484)
Supplement: Supplementary file 2 [file Table_2.DOCX]

**Appendix 2**

**High Performance Liquid Chromatography (HPLC) Analysis of HQT Decoction**

**Method:**

Sample preparation: 183g HQT formula macerated in 2500ml deionized water for 0.5h before two times boiling. After filtration, the filtrates were merged and centrifuged at 3000rpm for 5min. Finally, the supernatant was concentrated to 1g/ml. To prepare sample for high performance liquid chromatography (HPLC), 100 μl of sample was dissolved in 1 ml methanol and filtered. Agilent 1260LC series HPLC system (Agilent Technologies, USA) was performed in this study for qualitative analysis of HQT formula. The working condition was optimized and established as follows: separation was achieved on a ZORBAX SB-C18 (4.6×250 mm, 5 μm) column by using water (A) and acetonitrile (C) as the mobile phase. The gradient elution setting listed as follows: 0 min, 90% A+10%C; 0-2 min, 90%A+10%C→ 99%A+1%C ; 2-8 min, 99% A+1%C → 100% A+0%C; 8-15 min, 100% A+0%C → 60% A+40%C; 15-35 min, 60% A+40%C → 30% A+70%C; 35-45 min, 30% A+70%C → 10% A+90%C; 45-55 min, 10% A+90%C → 90% A+10%C. The flow rate was 0.7 ml/min and the injection volume was 10ul. Ultraviolet spectra in 212 nm was used for detection. Ten standard solutions (one standard solution for one herb in HQT formula) showed in table 1 were used for qualitative analysis.

| Table 1 \| Ten phytochemicals identified in HQT. | | |
| --- | --- | --- |
| Peak number | Herbal | Identification |
| 1 | Salvia miltiorrhiza Bunge | Tanshinone IIA |
| 2 | Dioscorea nipponica Makino | Dioscin |
| 3 | Astragalus membranaceus | Astragaloside A |
| 4 | Paeonia tacti lora Pall | Paeoniflorin |
| 5 | Saussurea involucrata (Kar. et Kir.) Sch.-Bip | Rutin |
| 6 | Eucommia ulmoides Oliver | Pinoresinol Diglucoside |
| 7 | Davallia mariesii Moore ex Bak | Naringin |
| 8 | Dipsacus asperoides C. Y. Cheng et T. M. Ai | Asperosaponin Ⅵ |
| 9 | Chinese Foxglove | Acteoside |
| 10 | Glycyrrhizauralensis | Glycyrrhizic Acid |

**Result:**

Chromatogram of eight chemical compositions in HQT was depicted in Fig. 1. Two of ten, Tanshinone Ⅱ and Saponins, couldn’t identify. The reason for loss signal of Tanshinone Ⅱ is it will become unstable after two times boiling. And the peak of Yam saponins is too close with Astragalus glycosides to be identified. So, in total, only eight of them can be found in the water extract, which were conducted in a stable quality.


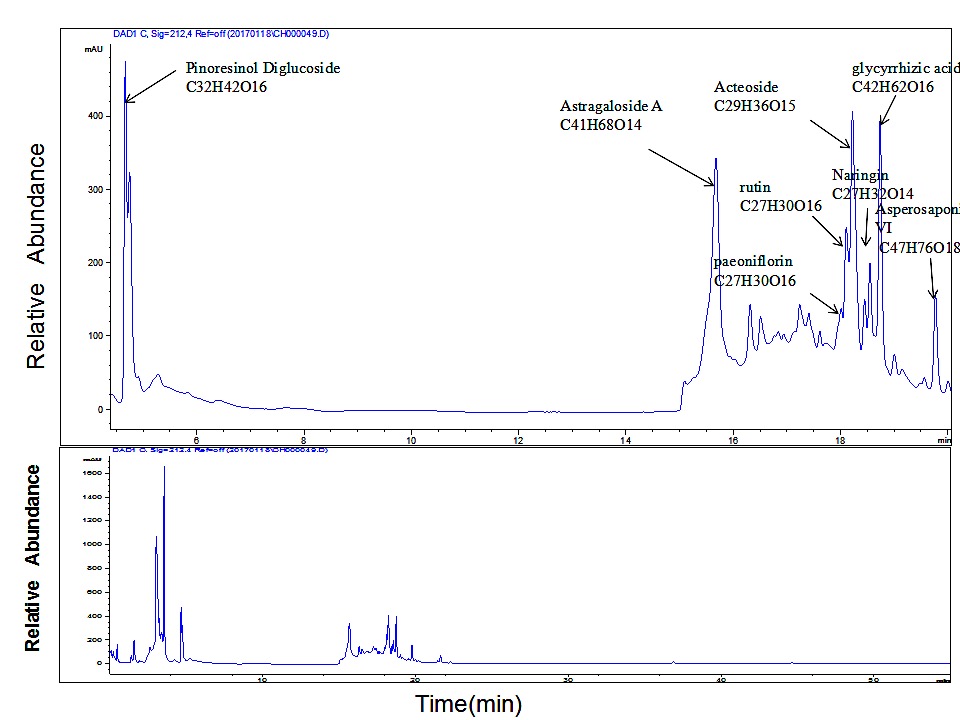


Fig. 1 The base peak ion chromatogram of the HQT decoction analyzed by HPLC.

Fig. 2 The base peak chromatogram of the phytochemicals in HQT.

Fig. 3 The base peak of the Dioscin in HQT. (tR=15.692 min)

Fig. 4 The base peak of the Astragaloside A in HQT. (tR=15.712 min)

Fig. 5 The base peak of the Paeoniflorin in HQT. (tR=18.012min)

Fig. 6 The base peak of the Rutin in HQT. (tR=18.105min)

Fig. 7 The base peak of the Pinoresinol Diglucoside in HQT. (tR=4.489 min)

Fig. 8 The base peak of the Naringin in HQT. (tR=18.67 min)

Fig. 9 The base peak of the Asperosaponin Ⅵ in HQT. (tR=19.761 min)

Fig. 10 The base peak of the Acteoside in HQT. (tR=18.28 min)

Fig. 11 The base peak of the Glycyrrhizic Acid in HQT. (tR=18.623 min)
