# Supplementary material for: Chinese Herbal Formula Huayu-Qiangshen-Tongbi Decoction Compared With Leflunomide in Combination With Methotrexate in Patients With Active Rheumatoid Arthritis: An Open-Label, Randomized, Controlled, Pilot Study
Source: Front Med (Lausanne). 2020 Sep 4;7:484. doi: 10.3389/fmed.2020.00484 (PMC7498571; doi:10.3389/fmed.2020.00484)
Supplement: Supplementary file 3 [file Table_3.DOCX]

**Appendix 3**

**Supplementary table 1.** Clinical and laboratory measures of the two groups at each visit in the PP analysis.

| **Measures** | | **Time** | | | **MTX+HQT** | **MTX+LEF** | ***P* value**  **(within the group)** | | ***P* value**  **(between groups)** |
| --- | --- | --- | --- | --- | --- | --- | --- | --- | --- |
|  |  |  |  |  |  |  | **MTX+HQT** | **MTX+LEF** |  |
| **TJC, n** | | **0w** | 8.82 (5.15) | | | 8.57 (5.54) |  |  | 0.606 |
|  |  | **4w** | 6.49 (4.86) | | | 5.78 (4.35) | 0.011 | <0.001 | 0.465 |
|  |  | **12w** | 4.51 (3.43) | | | 4.41 (3.82) | <0.001 | <0.001 | 0.691 |
|  |  | **24w** | 3.23 (3.91) | | | 2.68 (3.56) | <0.001 | <0.001 | 0.317 |
| **SJC, n** | | **0w** | 6.23 (4.12) | | | 4.49 (3.14) |  |  | 0.042 |
|  |  | **4w** | 4.13 (3.39) | | | 2.81 (2.89) | 0.007 | 0.001 | 0.073 |
|  |  | **12w** | 1.90 (2.29) | | | 1.89 (2.55) | <0.001 | <0.001 | 0.741 |
|  |  | **24w** | 1.69 (3.86) | | | 0.92 (1.72) | <0.001 | <0.001 | 0.656 |
| **Patient’s assessment of pain, mm** | | **0w** | 64.85 (17.23) | | | 60.54 (19.71) |  |  | 0.322 |
|  |  | **4w** | 46.92 (18.38) | | | 43.38 (21.08) | <0.001 | <0.001 | 0.313 |
|  |  | **12w** | | 30.77 (21.57) | | 30.62 (19.73) | <0.001 | <0.001 | 0.900 |
|  |  | **24w** | | 23.15 (19.00) | | 20.59 (17.37) | <0.001 | <0.001 | 0.572 |
| **PaGADA, mm** | | **0w** | | 61.79 (15.37) | | 56.35 (19.03) |  |  | 0.229 |
|  |  | **4w** | | 42.18 (20.42) | | 43.78 (20.73) | <0.001 | <0.001 | 0.735 |
|  |  | **12w** | | 29.49 (19.32) | | 30.95 (20.68) | <0.001 | <0.001 | 0.725 |
|  |  | **24w** | | 20.77 (17.07) | | 19.86 (18.20) | <0.001 | <0.001 | 0.682 |
| **PhGADA, mm** | | **0w** | | 62.05 (19.08) | | 58.38 (20.35) |  |  | 0.424 |
|  |  | **4w** | | 40.18 (21.45) | | 45.14 (21.81) | <0.001 | <0.001 | 0.321 |
|  |  | **12w** | | 29.74 (20.45) | | 29.46 (20.13) | <0.001 | <0.001 | 0.929 |
|  |  | **24w** | | 19.18 (17.84) | | 19.32 (18.78) | <0.001 | <0.001 | 0.928 |
| **Morning stiffness, min** | | **0w** | | 45.13 (27.23) | | 48.78 (43.69) |  |  | 0.485 |
|  |  | **4w** | | 27.95 (29.06) | | 35.41 (43.58) | 0.001 | 0.008 | 0.624 |
|  |  | **12w** | | 18.38 (34.07) | | 14.86 (18.95) | <0.001 | <0.001 | 0.781 |
|  |  | **24w** | | 20.00 (51.41) | | 11.24 (23.57) | <0.001 | <0.001 | 0.277 |
| **CRP, mg/L** | | **0w** | | 19.90 (19.60) | | 27.99 (37.51) |  |  | 0.499 |
|  |  | **4w** | | 15.50 (19.81) | | 15.06 (26.91) | 0.007 | 0.001 | 0.448 |
|  |  | **12w** | | 13.57 (15.74) | | 19.65 (60.50) | 0.003 | <0.001 | 0.277 |
|  |  | **24w** | | 12.49 (19.03) | | 10.10 (17.73) | 0.001 | <0.001 | 0.323 |
| **ESR, mm/h** | **0w** | | | 62.51 (25.86) | | 54.35 (30.50) |  |  | 0.211 |
|  | **4w** | | | 56.87 (27.17) | | 49.05 (31.66) | 0.061 | 0.035 | 0.165 |
|  | **12w** | | | 53.23 (31.68) | | 42.11 (28.80) | 0.068 | <0.001 | 0.151 |
|  | **24w** | | | 47.82 (27.95) | | 39.76 (27.35) | 0.001 | 0.001 | 0.205 |
| **RF, U/mL** | **0w** | | | 209.51 (293.98) | | 183.68 (205.13) |  |  | 0.913 |
|  | **4w** | | | 159.15 (230.96) | | 187.28 (261.46) | 0.003 | 0.034 | 0.758 |
|  | **12w** | | | 151.44 (230.44) | | 122.62 (211.60) | <0.001 | 0.002 | 0.695 |
|  | **24w** | | | 227.20 (456.20) | | 110.41 (148.24) | 0.100 | 0.001 | 0..633 |
| **HAQ** | **0w** | | | 0.63 (0.57) | | 0.89 (0.68) |  |  | 0.110 |
|  | **4w** | | | 0.47 (0.46) | | 0.57 (0.59) | 0.009 | <0.001 | 0.590 |
|  | **12w** | | | 0.37 (0.48) | | 0.42 (0.45) | <0.001 | <0.001 | 0.306 |
|  | **24w** | | | 0.23 (0.38) | | 0.26 (0.45) | <0.001 | <0.001 | 0.779 |
| **DAS28-CRP** | **0w** | | | 6.13 (1.85) | | 5.80 (2.07) |  |  | 0.316 |
|  | **4w** | | | 4.80 (2.07) | | 4.29 (1.90) | <0.001 | <0.001 | 0.180 |
|  | **12w** | | | 3.67 (1.61) | | 3.53 (1.73) | <0.001 | <0.001 | 0.647 |
|  | **24w** | | | 3.14 (1.78) | | 2.75 (1.59) | <0.001 | <0.001 | 0.197 |

* Values are the mean,SD. TJC, tender joint count; SJC, swollen joint count; PaGADA, patient’s global assessment of disease activity; PhGADA, physician’s global assessment of disease activity; HAQ, Health Assessment Questionnaire; ESR, erythrocyte sedimentation rate; CRP, C-reactive protein; RF, rheumatoid factor; DAS28, 28-joint disease activity score.

† Measured on a 100-mm visual analog scale.
